# Colour Vignetting Correction for Microscopy Image Mosaics Used for Quantitative Analyses

**DOI:** 10.1155/2018/7082154

**Published:** 2018-06-07

**Authors:** Filippo Piccinini, Alessandro Bevilacqua

**Affiliations:** ^1^Istituto Scientifico Romagnolo per lo Studio e la Cura dei Tumori (IRST) IRCCS, Meldola, Italy; ^2^Advanced Research Center on Electronic Systems for Information and Communication Technologies “E. De Castro” (ARCES), University of Bologna, Bologna, Italy; ^3^Department of Computer Science and Engineering (DISI), University of Bologna, Bologna, Italy

## Abstract

Image mosaicing permits achieving one high-resolution image, extending the visible area of the sample while keeping the same resolution. However, intensity inhomogeneity of the stitched images can alter measurements and the right perception of the original sample. The problem can be solved by flat-field correcting the images through the vignetting function. Vignetting correction has been widely addressed for grey-level images, but not for colour ones. In this work, a practical solution for the colour vignetting correction in microscopy, also facing the problem of saturated pixels, is described. In order to assess the quality of the proposed approach, five different tonal correction approaches were quantitatively compared using state-of-the-art metrics and seven pairs of partially overlapping images of seven different samples. The results obtained proved that the proposed approach allows obtaining high quality colour flat-field corrected images and seamless mosaics without employing any blending adjustment. In order to give the opportunity to easily obtain seamless mosaics ready for quantitative analysis, the described vignetting correction method has been implemented in an upgraded release of* MicroMos* (version* 3.0*), an open-source software specifically designed to automatically obtain mosaics of partially overlapped images.

## 1. Introduction

Panoramic photography is very popular. It has been used for more than a century; the first attempts of panoramic photographs are found at war photography (*e.g.,* during the American Civil War in 1860) [1]. Nowadays, they are widely used in oncology, in particular in histopathology [2]. As far as microscopy is concerned, various high-magnification microscopes are used in order to observe the fine details of biological specimens. However, they all suffer from a limited field of view (FOV) [3]. Mosaicing has a key role for this purpose, since high-resolution images representing a whole sample are a valuable resource for pathologists and biologists in general [4]. Technically,* image mosaicing* is defined as the process of obtaining a wider FOV of a scene from a tile of partial views, and* mosaics* are built by registering and stitching several overlapping images [5]. In order to obtain a mosaic suitable for quantitative analysis, two important aspects must be considered: the geometric alignment of the images and the colour homogenization of the different views [6]. Errors propagated via geometric and photometric misalignments result in undesirable seams and object discontinuities that can be then seen at the borders of the images.

Ghosh and Kaabouch provided an in-depth survey of the existing image mosaicing algorithms, by classifying them into several categories [7]. Furthermore, a number of image mosaicing algorithms have been proposed in the literature over the last three decades [8]. For instance,* Autostich* [9] and* Image Composite Editor* (http://research.microsoft.com/en-us/um/redmond/groups/ivm/ICE/) are two very popular freely available software tools widely used off-line to stitch partly overlapping images. On the one hand, the geometric point of view has been thoroughly investigated by the computer vision community [10]. On the other hand, colour mapping (*i.e.,* correction of colour mismatches) used to obtain seamless mosaics for quantitative analyses has not been extensively studied yet and the tonal registration of colour images is still a pending problem.

**Figure 1:**
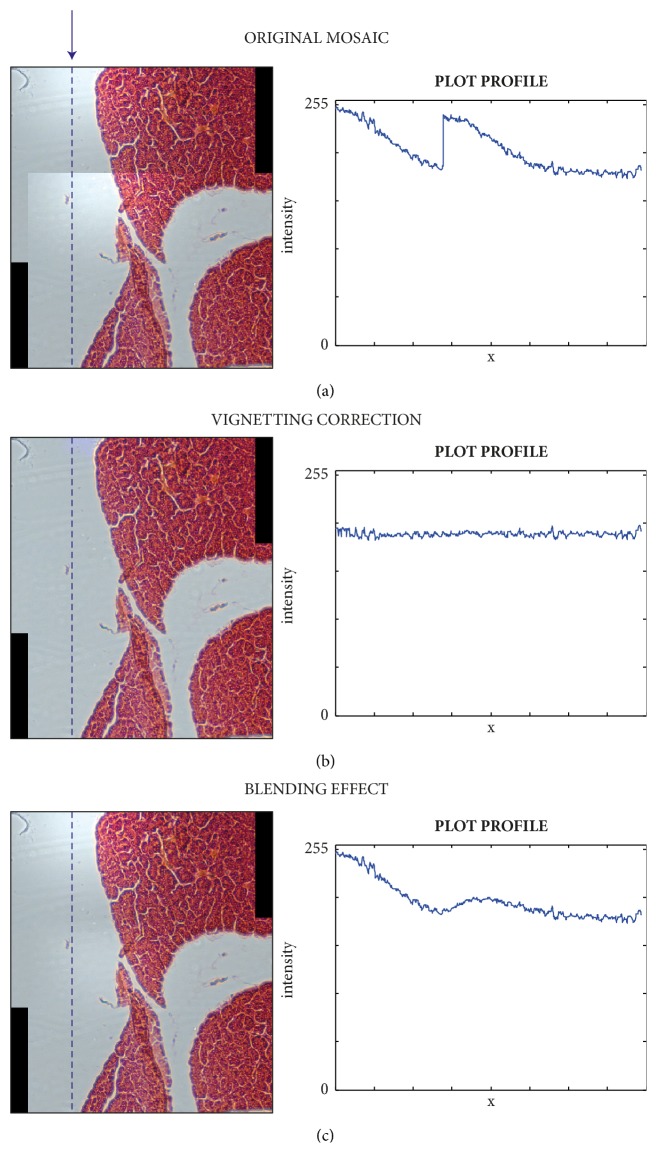
Vignetting correction versus blending. (a) Mosaic obtained by stitching together two partially overlapped images, without performing any tonal correction. On the right, the intensity plot profile of the dashed-blue line highlighted in the mosaic on the left. (b) Same mosaic as (a) but obtained by vignetting correction. The intensity profile became flat. (c) Same mosaic as (a) but obtained by minimizing the discontinuities in the stitching zones through blending. Globally, intensity discontinuities still remain in the mosaic as shown by the plot profile.

Microscope image acquisition has several limitations due to imperfect illumination of the specimen, optical aberrations in the objectives, and different sources of camera noise [11]. These conditions can cause the generation of images with inhomogeneous intensity, a phenomenon generally referred to as* vignetting* [12]. As such, vignetting reduces overall intensity of objects in the periphery or other parts of the image [13], while noise is increased [6]. In practice, uneven distribution of the FOV's intensity is often tolerable if images are analyzed qualitatively. On the contrary, when quantitative measurements are needed, uneven illumination hides real quantitative differences and jeopardizes biological experiments [14]. Although blending is used to minimize the discontinuities along the stitching regions of mosaics [7], it cannot fix the problem of inhomogeneous brightness; hence discontinuities remain in the mosaic (Figure 1). Flat-field correction performed using the vignetting function is the only solution available to obtain seamless mosaics with undistorted intensity values [15].

Several linear [16] and nonlinear [17] vignetting correction approaches have been proposed in literature for grey-level images and the problem has been extensively discussed [18]. For instance, Liu* et al*. [19] deeply compared 10 different approaches and Peng* et al*. [20] tested the most recent software implementations using 12 microscope image collections (also provided for future analyses). Chernavskaia* et al*. [21] wrote an extensive tutorial article with practical recommendations for the application of different flat-field correction methods to the development of automatic software for medical diagnostics. However, the literature about vignetting correction of colour images is very sparse [22].

In this work: (a) an efficient colour vignetting correction approach for microscopy images is described, also tackling the problem of under- and overexposure [15]; (b) a new release of* MicroMos* (http://sourceforge.net/p/micromos) [23], a software recently proposed to automatically obtain mosaics of partially overlapped images, is presented. In particular, four new modules and a Graphical User Interface (GUI) have been added, which make the software user friendly; (c) mosaics obtained with different tonal correction approaches are quantitatively compared by using state-of-the-art metrics. To quantitatively compare the different correction approaches, we exploited the registration matrices directly provided by* MicroMos* as the output. It is worth noticing that identifying corresponding pixels to quantitatively compare original images and related mosaics would be not possible without using software that provides the registration matrixes used to build the mosaics. In this way* MicroMos*, a software tool conceived for mosaicing can also be used as a tool to compare registration approaches.

## 2. Materials and Methods

### 2.1. Colour Vignetting Correction

The microscope endowed with a digital camera represents an image acquisition system with a single illumination source, with constant properties for a long acquisition time. A sample imaged at different positions produces different scenes but the corresponding RGB pixels are related by simple scale factors [24]. Consequently, the flat-field correction performed by using the vignetting function (also called retrospective correction [25]) enables normalizing the image's intensities by providing homogeneous pixels' values representative of the original sample's radiance.

The flat-field correction theory is complex [18]. Several methods have been proposed in literature to estimate the vignetting function from a single-image [26] or a sequence of images acquired by keeping the microscope set-up constant [12]. The most common way to do that is using a short sequence of empty field images and computing the median value for each* x*-*y* pixel position [27]. Other approaches rely on the segmentation of the background [28] or foreground [29] regions followed by a dense 2D reconstruction. However, once the vignetting function* V* has been estimated, each grey-level pixel of the input image* I* is then flat-field corrected by simply normalizing the intensities according to (1) (see [30]). Consider

(1) $$\begin{array}{c} O\left(\;x,y\;\right)=\frac{I\left(x,y\right)}{V\left(x,y\right)}\bar{V} \end{array}$$

where $\bar{V}$ is the mean value of *V*, (*x*, *y*) represent the 2D pixel's coordinates, and *O* is the flat-field corrected image. In the literature, there are also many works showing colour mosaics flat-field corrected [31]. However, most of the authors do not declare which colour space they use, neither which channel is modified to flat-field correct the images, and only few of them give details on how they transfer the theory of vignetting correction from grey-level images to colour ones [15].

Vignetting is basically a channel independent effect [32]; the red, green, and blue channels in an RGB image are all affected by the same function and to correct vignetting all channels are typically multiplied by the same correction factors [33]. Accordingly, Sun* et al*. [34] calculated the average intensity from all channels and corrected colours image with the same approach used for monochrome ones. In this work, the vignetting function is the median value for each* x-y* position of empty field images converted into grey-level and stored into a* z*-stack. Then, similarly to what Kordecki* et al*. [35, 36] described, we proposed a colour vignetting correction approach where* V* is used to normalize each channel *c* ∈ {R, G, B} as reported in

(2) $$\begin{array}{c} O\left(\;x,y,c\;\right)=\frac{I\left(x,y,c\right)}{V\left(x,y\right)}\bar{V}. \end{array}$$

**Figure 2:**
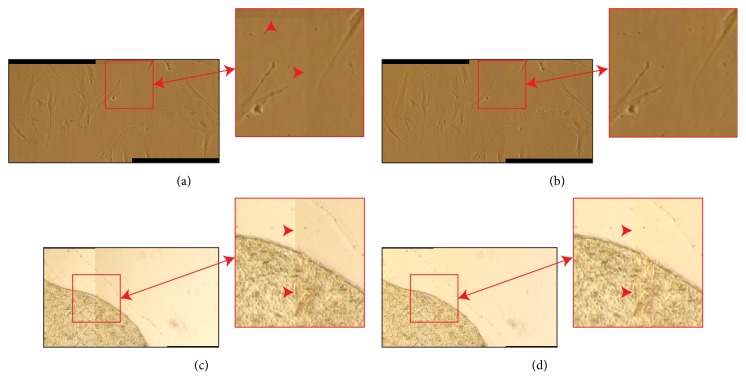
Colour mosaics of cells and tissues. (a, c) Mosaics obtained by stitching two partly overlapping images, without performing any tonal correction. On the right, a high-magnification detail representing a region across the stitching seam (highlighted with red arrowheads in the magnified details). The images in (a) do not have saturated pixels, while those in (c) have pixels saturated in the red channel. (b, d) Same mosaics as (a) and (c), respectively, but obtained by vignetting correcting the images according to (2). Seams in the stitching zones are still visible in (d).

Then, for each* c* of* O*, the underexposed and the saturated pixels originally present in* I*(*x,y,c*) are remapped back to* 0* and the maximum intensity value (usually,* 255*). This simple yet effective approach enables obtaining seamless mosaics, except when saturated pixels are present in at least one* c* (Figure 2).

### 2.2. Pixel Saturated Images

**Figure 3:**
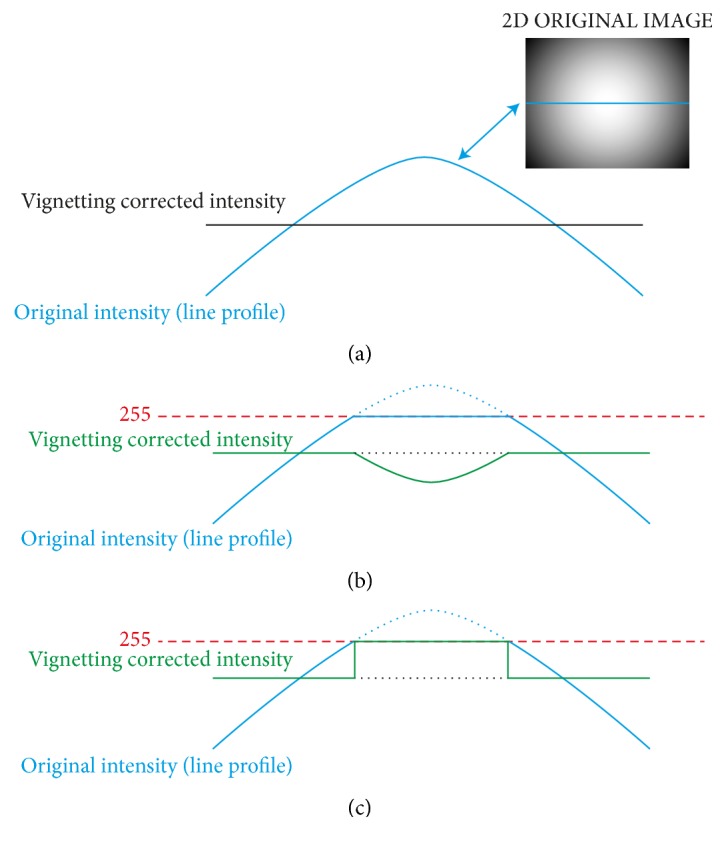
Saturated pixels. (a) Let us consider the blue continuous curve as a representative of the intensity profile of the blue line in the image reported in the top-right corner. Considering the image as directly proportional to the vignetting function, the black continuous line represents the intensity profile after correcting vignetting according to (1). (b) All the saturated pixels (represented as a blue dotted segment), pixels with intensity value higher than* 255* (red dashed line), are captured at* 255*, thus losing the connection with the original radiance. The same happens for the green continuous curve achieved after normalizing the new blue continuous curve with the vignetting function. (c) In the proposed vignetting correction approach, all pixels that before normalization were at* 255* are set back to* 255*.

In order to maximize the visual contrast on the samples, a great quantity of light is required, through a wide iris aperture or, less frequently, through a high exposure time. As a drawback, several parts of the images (the whitest ones) can go towards saturation or even saturate. The problem of pixels with values at the range limits is typically neglected by authors presenting colour-correction approaches [37]. Only few papers describe what procedure is implemented to deal with saturated pixels [38]. It is worth remarking that the digital images represent the scene's radiance using a limited colour depth, usually* 8* bits. Very low and high radiance values are not accurately represented in the image [15]. Accordingly, recovering the real sample's radiance from underexposed or saturated images without using prior information is impossible. Consequently, false-colours (meant as colours not representing the real sample's radiance) are artificially generated if saturated images are flat-field corrected considering the saturated pixels in the same way as the “good” pixels [10], that is, the nonsaturated ones (Figure 3). In addition, if the images are used for quantitative analyses, the intensity values generated by normalizing the saturated pixels with the vignetting function can produce information (*e.g.,* unnatural profiles in tissues, as the green curve in Figure 3(b)) that lead to wrong conclusions.

In the proposed vignetting correction approach, all pixels originally at a* 255*-value after normalization are set back to* 255* (Figure 3(c)). Similarly, all pixels with* 0*-value before normalization are kept to* 0* also after vignetting correction. Although this approach does not provide a solution to recover the sample's radiance from saturated pixels, it does not introduce any false-colour in the corrected image.

**Figure 4:**
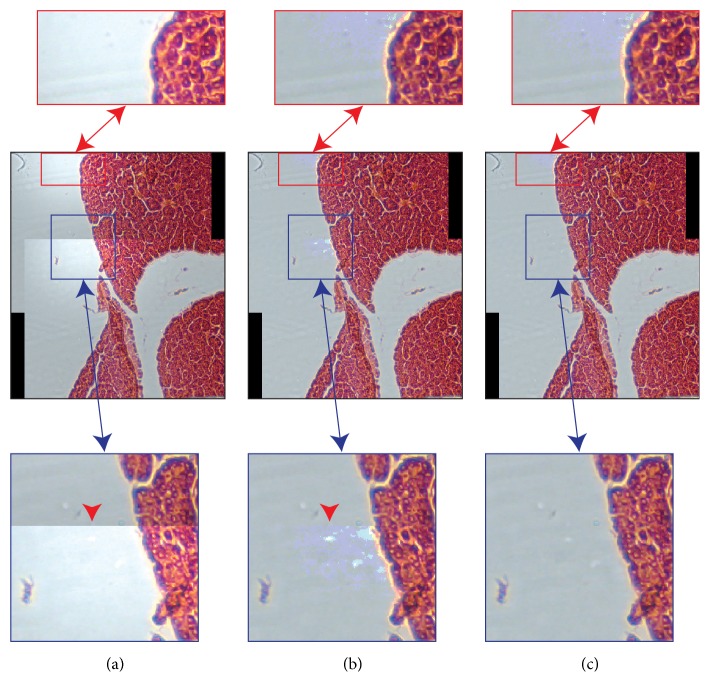
Mosaics of two partially saturated images. The saturated pixels in the overlapping region of the first image are not saturated in the second one. The details reported in the red boxes show a region where the two stitched images do not overlap, while the details in the blue boxes show an overlapping region. (a) Mosaic obtained by stitching the images without performing the vignetting correction. (b) Mosaic obtained through vignetting correcting the images according to (2). Discontinuities generated by the presence of saturated pixels are visible in the two magnified details (highlighted also from arrowheads). (c) Mosaic obtained by vignetting correcting the images according to the OUSPC approach explained in Section 2.2. The discontinuities, previously shown in the blue boxes, disappeared because in correspondence of that overlapping part there are unsaturated pixels available in the other image. Instead, the discontinuities highlighted in the red boxes (*i.e., *where the two stitched images do not overlap) are still visible.

In mosaicing applications, more representations of the same scene are available, and pixels that are underexposed/saturated in some images might not be underexposed/saturated in other ones, due to the spatial nonuniformity of the system [5]. Accordingly, a way to recover the sample's radiance from underexposed/saturated pixels can be exploiting the overlapping areas and, for each channel* c* and pixel* p* of the flat-field corrected image* O*_i_ (with* i*=*1.*..*n*,* n* = number of images composing the mosaic), checking whether “good” intensity values* v* contained in the correct range (*i.e., 0* <* v* <* 255*) are present in the corresponding* p* of the other flat-field corrected images* O*_j_, with* j≠i* (it is worth noting that* corresponding p* in different* O* have always a different (*x,y*) position, except when* O*_i_ and* O*_j_ are perfectly geometrically aligned). In that case, the underexposed/saturated intensity of the pixels in* O*_i_, corresponding to the original underexposed/saturated pixels in* I*_i_, can be replaced by the corresponding “good” flat-field corrected intensity values taken from the other flat-field corrected images* O*_j_ (Figure 4). Accordingly, the core of the proposed algorithm can be expressed in pseudocode as follows

 
**for each** channel* c* of* O*_*i*_
   
**for each** pixel* p* of* O*_*i*_
     
**if*** O*_*i*_(*p,c*)=255 or* O*_*i*_(*p*,*c*)=0
       
**if** image* O*_*j*_ exists with* O*_*j*_(*p*,*c*)≠255 and* O*_*j*_(*p*,*c*)≠0
         
*O*_*i*_(*p*,*c*)*←**O*_*j*_(*p*,*c*)

We named this approach Overlapping-based Underexposed/Saturated Pixels Correction (OUSPC) because it is worth noting that this tonal correction works in the cases where more images of the same scene are available (*e.g.,* mosaicing applications). Furthermore, this approach requires a perfect geometric registration and it fixes the problem of underexposed and saturated pixels only in the overlapping parts of the images (*e.g.,* the detail shown in the blue bounding boxes of Figure 4(c)). Consequently, it could generate discontinuities (*e.g.,* colour fringes [39]) outside the overlapping regions (see the details shown in the red bounding boxes of Figures 4(b) and 4(c)).

### 2.3. Blending Technique

**Figure 5:**
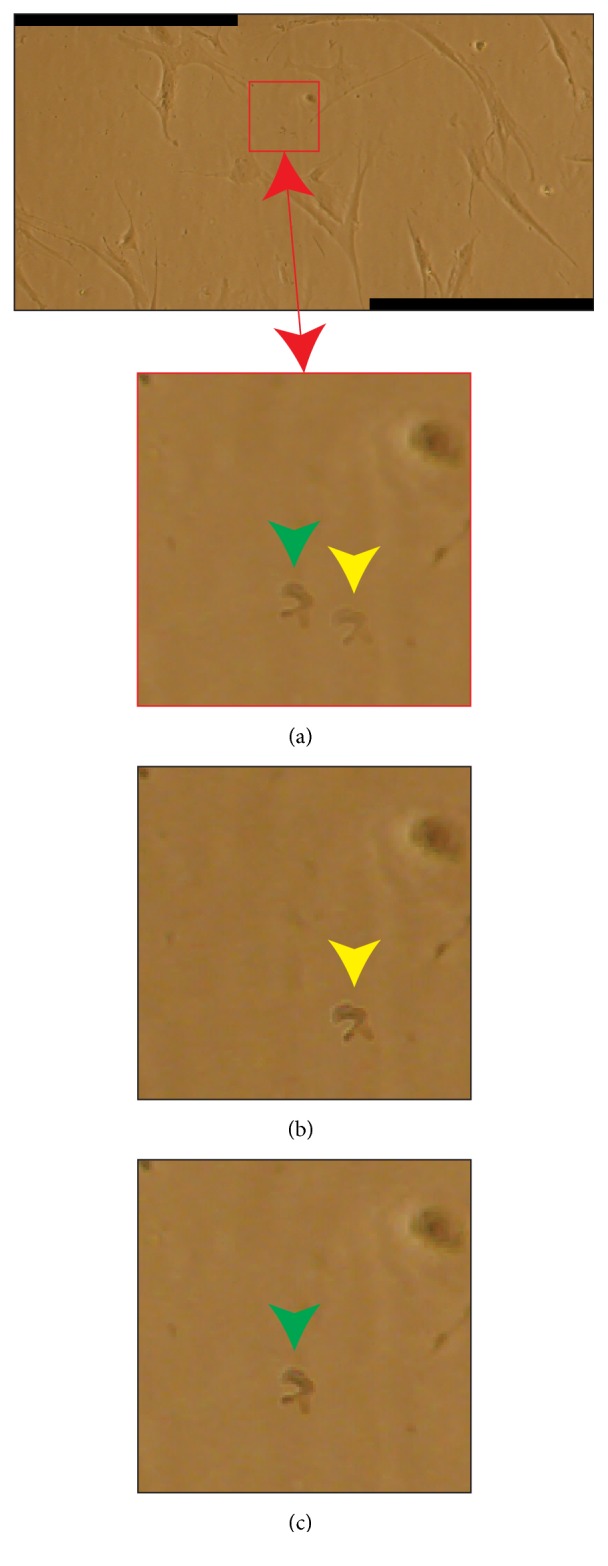
Ghost effect due to blending weighting. (a) Mosaic of two partially overlapping images representing a monolayer culture of living cells. Corpuscles and debris floating in the culture medium generate blur objects that look like shadings, when blending is used. In red colour, a magnification detail showing a corpuscle artificially duplicated as a side effect of blending. (b, c) Same detail reported in a red squared, but coming from the two original images composing the mosaic. The corpuscle in motion is present once only for each original image, as shown by the green and yellow arrowheads.

**Figure 6:**
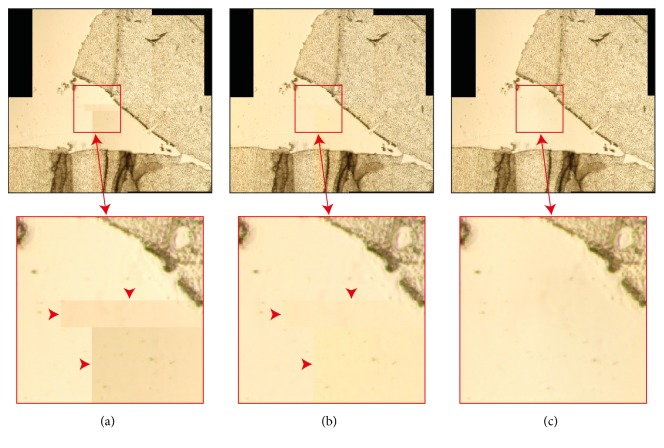
Mosaics of fully saturated images. In the overlapping parts, all the stitched images show pixels saturating in the red channel. (a) Mosaics obtained by stitching four partially overlapped images without performing the vignetting correction. (b) Mosaic obtained by vignetting correcting the images according to Eq. (2). Seams in the stitching zones are still visible (highlighted also from arrowheads). There are no differences with the mosaic built according to the OUSPC approach explained in Section 2.2, because in the overlapping parts all the images of this set show saturated pixels after flat-field correction. (c) Mosaic obtained without performing the flat-field correction, but using the blending approach described in Section 2.3. No seams are visible.

Employing blending techniques is the only way to obtain fully seamless mosaics if all the original images have pixels with underexposed and saturated values in the parts to be overlapped. Piccinini* et al.* [40] have recently proposed a blending solution based on a bilinear interpolation of the intensity values of the pixels in the overlapping regions of the images to be stitched. The values of pixels within a transition zone are computed through a weighted average of the relative pixel values in the different images [41]. First of all, the overlapping region* OR* between the mosaic* M* and the new image* I* to be stitched is computed by estimating the bounding-box coordinates according to registration matrix. Then, a weighting mask* M*, with dimension equal to* OR* and values in the range [0, 1], is used for the final stitching of* I*. The values of the inner pixels of* M* are computed by considering the Euclidean distances between the inner pixel and the closer border of* OR*, differentiating between borders belonging to* M* or* I*. For the sake of clarity, to make the reader better understand the proposed approach, the authors provided an algorithm in pseudocode, also describing in detail how to process colour images. It is worth noting that blending approaches require a perfect geometric registration; otherwise “ghost” objects (*i.e., *duplicate blur objects, visible as shadings Figure 5) may appear [42]. However, exposure differences, discontinuities in the border of the overlapping regions, and seams in the stitching zones disappear by using a bilinear blending method to fade the images in the overlapping area [43] (Figure 6). Blending can actually address efficiently the problem if the goal is to obtain a good-looking beautiful mosaic only for aesthetic reasons, without discontinuities in the stitching zones. However, discontinuities still remain in the mosaics (*e.g.,*Figure 1(c)), and this is the reason why they should not be used for quantitative analyses.

### 2.4. *MicroMos* Version 3.0

*MicroMos* is a software tool specifically designed to automatically stitch together a tile of partially overlapping microscopy images. Briefly, the Shi-Tomasi [44] corner detector is used to extract salient points for each pair of subsequent images, and the LKT tracker [45] is then used to determine correspondences between corners at subpixel accuracy. Various warping models (*i.e.,* translational, affine, and projective) and registration approaches (*i.e.,* frame-to-frame and frame-to-mosaic) are available. Finally, a single full-resolution mosaic is saved as output of the process.

An early version of* MicroMos* (version* 1.0*), able to operate with images acquired with label-free microscopy techniques only, was proposed in [23]. The software was then extended to operate with fluorescent images, providing a specific module to correct for intensity decay due to photobleaching effects (*MicroMos v2.0*, [46]). Finally, a new registration strategy, based on the phase-correlation algorithm [47], was implemented to obtain mosaics of images characterized by highly repeated patterns such as the images of a hemocytometer's grid [40].

**Figure 7:**
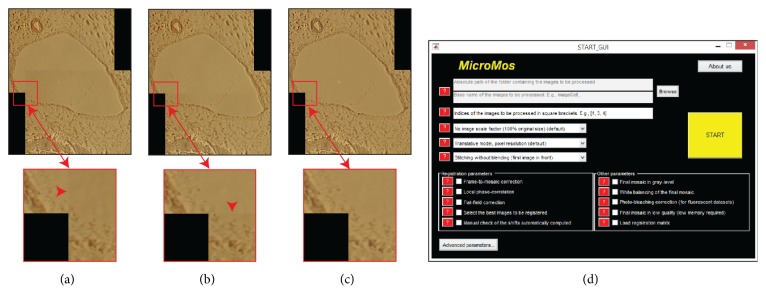
*MicroMos* GUI. The new release of* MicroMos* is endowed with a GUI that makes the selection of the different modules, to choose different geometric and tonal registration strategies, very simple. (a) Colour mosaic obtained without performing any tonal correction and imposing the first registered image into the front (*i.e.,* upper layer). (b) Same mosaic as (a), but with the last image into the front. Seams (highlighted also from arrowheads) are visible in the magnified details. (c) Same mosaic as (a), but vignetting corrected. No seam is now visible in the high-magnification detail reported at the bottom of the mosaic. (d) GUI of* MicroMos* with the main screen describing all the buttons and flags that enable the function of the various modules.

In this new release of* MicroMos* (version* 3.0*), four new modules were implemented. (*I*) The first one enables a manual correction of the image alignments automatically estimated. (*II*) The second module enables determination of the order of overlapping (*i.e.,* layers) between subsequent images, choosing between “first image stitched in front” (*i.e.,* first image shown in the upper layer, Figure 7(a)) and “last image stitched in front” (*i.e.,* first image shown in the bottom layer, Figure 7(b)). The first option is useful to work with fluorescent datasets so as to have a mosaic representative of the original sample status before intensity decay due to the photobleaching effect. (*III*) The third module enables flat-field correcting the images according to the method described in Section 2.1 (Figure 7(c)). Furthermore, an additional parameter enables the OUSPC colour remapping strategy introduced in Section 2.2. (*IV*) Finally, it is now possible to load an external registration matrix, to enable the user to register the images using different tonal corrections methods, while keeping the geometric shifts unchanged. Furthermore,* MicroMos v3.0* has been endowed with a Graphical User Interface (GUI), where every module is now coupled with a help menu, and several flags enable the function of the different modules (Figure 7(d)).

*MicroMos* is written in MATLAB (The MathWorks, Inc., Massachusetts, USA). Source code and standalone executable version (*i.e.,* not requiring MATLAB being installed) are freely distributed as an open-source software tool at http://sourceforge.net/p/micromos.

## 3. Results

### 3.1. Image Datasets

In order to assess the quality of the proposed vignetting correction approach, seven pairs of partially overlapping images of seven different samples were acquired. The datasets were acquired in brightfield, or phase-contrast, by using two different widefield optical microscopes, equipped with* 8*-bit/channel RGB colour cameras. The first microscope was an inverted Nikon (Tokyo, Japan) Eclipse TE2000-U, equipped with a colour Nikon DXM1200 digital camera (*2*/*3*” CCD sensor, pixels of* 6.7μ*m side,* 640*×*512* resolution) and a Plan Fluor* 10*×/*0.30* Ph1 DLL ∞/*0.17* objective lens. The second is an Optika (Bergamo, Italy) B-353-PLi microscope, equipped with a Matrix Vision (Stuttgart, Germany) BlueFOX 221C colour camera (*1/3*” CCD sensor, pixels of* 4.65μ*m side,* 1024*×768 resolution) and an Optika E Plan* 4*×/*0.10* BF ∞/*0.17* objective lens. The acquired images regard two cancerous bone samples (hereinafter, BONEa and BONEb), PANCREAS, STOMACH, TESTICLE histology specimens, a monolayer culture of living mesenchymal stromal cells (MSC), and a dead fruit fly (hereinafter, briefly FLY) held between a coverslip and a microscope slide.  Table 1 summarises the main features of the images used in the experiments. PANCREAS and FLY are characterized by a strong vignetting effect and, together with BONEa, are the only datasets showing saturated pixels.

In order to estimate the vignetting function, at the end of each acquisition stage a short sequence of empty field images was acquired by using the same equipment set-up. Briefly, the empty field images were converted into grey-level and stored into a* z*-stack. Then, the median value for each* x*-*y* position was computed and considered as* V*(*x,y*) value.

### 3.2. Quantitative Assessment

Once the images were acquired, the following five mosaics were compared for each dataset:

(a) Mosaic obtained without performing any tonal correction (hereinafter, briefly TA, standing for mosaic of “Type A”)

(b) Mosaic flat-field corrected according to (2) and remapping back to* 0* and* 255* the underexposed and the saturated pixels originally present, as explained in Section 2.1 (hereinafter, TB)

(c) Mosaic where vignetting is corrected according to the OUSPC approach proposed in Section 2.2 (TC)

(d) Mosaic obtained using the blending strategy outlined in Section 2.3 (TD), without any vignetting correction

(e) Mosaic obtained by performing both vignetting correction and blending (TE).

Comparing the mosaic with each input image is a common strategy widely used to assess the quality of a mosaic [48]. In this work, we adopted the strategy proposed in [23]. Briefly, each original image is first flat-field corrected and then warped and projected into the coordinate system of the mosaic using the corresponding registration matrix. In this way, it is easy to check the difference in intensity between corresponding pixels of the mosaic and the single composing images [23]. According to this strategy, for each pair of images used in the experiments, the second image registered was backprojected. It is worth noting that in the experiments performed in this work all mosaics were obtained by placing the first registered image into the upper layer. Accordingly, the first image was not backprojected (the difference in intensity would be* 0* for all pixels). Finally, considering only the pixels of the overlapping region, two standard parameters widely employed to measure the signal quality were computed.

First is Root Mean Squared Error (RMSE), defined according to

(3) $$\begin{array}{c} RMSE=\sqrt{\frac{\sum_{x}\sum_{y}{\left(OR\left(x,y\right)-BP\left(x,y\right)\right)}^{2}}{P}}. \end{array}$$

Second is Signal-to-Noise Ratio (SNR):

(4) $$\begin{array}{c} SNR=10\;\log_{10}\frac{\sum_{x}\sum_{y}OR{\left(x,y\right)}^{2}}{\sum_{x}\sum_{y}{\left(OR\left(x,y\right)-BP\left(x,y\right)\right)}^{2}}. \end{array}$$

In the above equations, (*x,y*) are the 2D pixel's coordinates;* OR* and* BP* point out the pixels of the overlapping-region of the mosaic and the overlapping part of the backprojected image, respectively;* P* is the number of pixels of* OR* and* BP*. In case signals are images, the image-specific Universal Quality Index (UQI, [49]) is also available and computed according to

(5) UQI=υOR,BPσOR·σBP·2·μOR·μBPμOR2+μBP2·2·σOR·σBPσOR2+σBP2

*μ*_OR_, *μ*_BP_, *σ*_OR_, *σ*_BP,_ and *υ*_(OR,BP)_ are mean, standard deviation (std), and covariance, respectively, of* OR* and* BP*. The UQI is defined mathematically and no human visual system model is explicitly employed. However, it has been widely proved in the literature to be able to measure the quality of images by “mimicking” what the human visual perception does [50]. For* 8*-bit images, RMSE ranges between* 0* and* 255*, where the lower the better. SNR and UQI range from [-∞, +∞] and [0, 1], respectively, where the higher the better.

### 3.3. Evaluation

**Figure 8:**
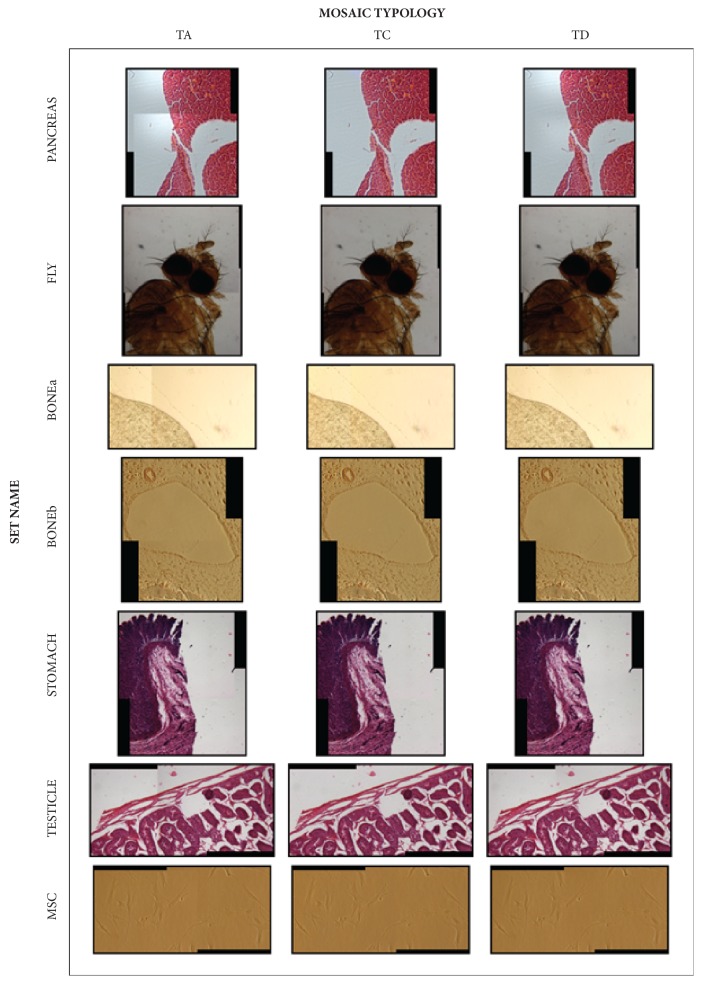
Set overview. In order to facilitate a comparison between the mosaics obtained with the proposed colour vignetting correction, TC mosaics are shown between TA and TD ones.

Figure 8 shows the TA, TC, and TD mosaics for each image dataset. TB and TE mosaics have been reported in Supplementary Figure 1 to keep mosaics in an appreciable resolution. As described in Section 2.2, TC mosaics are always as good as the TB ones or better. In particular, TC mosaics are better than TB if the registered images present underexposed or saturated pixels in the overlapping regions. Otherwise, the TC and the TB mosaics are the same. On the other hand, the TE mosaics are always the best for each dataset, because they present smoothed stitching regions, thanks to the blending, and they have no global intensity discontinuities, thanks to the vignetting correction.

As far as quantitative assessment is concerned, RMSE, SNR, and UQI were computed for each dataset and configuration by considering the pixels in the overlapping regions. The values are reported in Tables 2, 3, and 4, respectively. The values of the TE configuration are in italics, and for each dataset the best value obtained (the TE values aside) is in bold. RMSE and SNR values can be analyzed together because they present for each set the same rank. First of all, it is worth noting that the TA configuration showed the worst values for each metric, meaning that vignetting and blending always lead to improvement. For PANCREAS, FLY, and BONEa sets, which present saturated pixels in the overlapping region, TC mosaics were always better than the TB ones. For the other sets, TC and TB mosaics reached the same values.

With regard to RMSE and SNR, TC was better four times out of seven and TD three times. In particular, TC resulted as the best configuration for PANCREAS and FLY sets, characterized by a very strong vignetting effect and, for MSC, presenting living cells, corpuscles, and debris floating in the culture medium. It is worth remarking that, in case of moving objects, blending usually provides bad performances, due to the ghost side effect (Figure 5). For BONEa, STOMACH, and TESTICLE, characterized by a low vignetting effect, TD was the best configuration. However, considering the blending configuration as the best one for the sets characterized by a low vignetting effect would be a mistake. For instance, for BONEb, characterized by a low vignetting effect, TC resulted as the best configuration. Furthermore, by computing the absolute difference (AD) between RMSE values of TD and TC in Table 2, it can be seen that, in the three sets where TD had the lowest RMSE, AD was never higher than 12%. Meanwhile, in the four sets where TC was the best, AD was never lower than 34%. Similarly, the AD of SNR values reported in Table 3 for BONEa, STOMACH, and TESTICLE was never higher than 4% for SNR values, while for PANCREAS, FLY, MSC, and BONEb it was never lower than 8%. This means that when TD provides the best results, the TC configuration is similarly good. Oppositely, when TC is the best configuration, it is superior to TD.

Regarding the UQI, TC configuration performed the best two times out of seven. In particular, it was the best only for PANCREAS and FLY sets, characterized by a strong vignetting effect. As expected, the highest UQI values go to the blended mosaics, the purpose of which is to have a pleasant aspect, except for mosaics presenting a strong vignetting effect that causes global discontinuities, as highlighted in Figure 1(c).

However, it is worth noting that, for all the metrics, the TE configuration always resulted better than TD, meaning that the proposed colour vignetting correction always leads to improvement.

## 4. Discussion

Colour is an integral part of our visual world and one of the main image features used in art and photography [51]. In microscopy, most of the analyses of living cells and tissues are carried out by visualizing the samples in brightfield and phase contrast, and the corresponding RGB images are acquired using digital colour cameras. Many different colorimetric assays are used to analyze cell viability [52] as well as other morphobiological features [53] in the range of the visible light. For instance, Beachley* et al*. [54] performed cell counting for adhesion studies by analyzing RGB images of Alizarin Red-stained cells. Similarly, Masson's trichrome, a three-colour staining protocol, is widely used in histology to study at the same time the distribution of connective tissue (stained blue), nuclei (stained red/purple), and cytoplasm (stained red/pink) [55]. Therefore, colours are necessary to perform quantitative analyses [56] and different RGB triples, corresponding to different colours, may lead to the same grey-level conversion. Consequently, dedicated methods to correct vignetting in colour images must be adopted to obtain mosaics with homogeneous intensity, suitable for quantitative analyses.

Blending techniques are colour-mapping algorithms minimizing colour differences between views [57]. Many different colour-mapping approaches, also known as colour-registration, colour-correction, colour-balancing (if restricted to the overlapped area only), and colour-transfer [37], have been proposed in literature [58]. Their aim is to transfer the colour palette of the source image to the target one, while extending the transferred colour from the overlapped area to the full target image [59]. However, these approaches are prone to generate unnatural mosaics [60] and, in general, pseudocolours with pixels' intensity that is not representative of the original sample radiance. In practice, mosaics built by using blending images are typically good-looking and try effacing the seams but did not prevent them [5]. Accordingly, such mosaics cannot be used for quantitative analyses because the intensity values do not faithfully represent the original sample's radiance [61].

The mosaics generated with the proposed OUSPC approach overcame the ones achieved with the other methods by four out of seven times according to RMSE and SNR signal quality measures. Furthermore, when colour-correction was not the best, the related quantitative index values were always comparable with those of the method performing the best. Accordingly, the obtained results proved that the proposed solution for colour vignetting correction effectively allows creating seamless mosaics with undistorted intensity values, also recovering saturated pixels exploiting the overlapping regions of the mosaics.

As a future work, we have planned to perform vignetting correction experiments by using different image colour spaces, for instance, the Hue-Saturation-Intensity/Value (HSI/HSV). While, HSI and HSV do not provide solutions for restoration of saturated pixels, they permit optimizing the flat-field correction by considering the Intensity/Value channel only [62].

## 5. Conclusions

In this work, we proposed a practical colour vignetting correction for mosaicing applications. We also considered the problem of the out-of-range pixels: the original under- and the overexposed pixels are corrected by exploiting reliable values extracted from the same pixels in the overlapping images. In practice, in case a pixel is originally in saturation or becomes as such after vignetting correction, it is given the value it assumes in the first overlapping image where it is nonsaturated.

In the experiments performed, mosaics built with the proposed colour vignetting correction and other tonal correction strategies, including blending, were compared. To this purpose,* MicroMos v3.0* was employed, a software tool specifically designed for stitching overlapping images according to different selectable geometric and tonal registration strategies.

We proved that for each dataset the configuration employing the vignetting colour-correction and blending at the same time always resulted better than the configuration with blending only. This confirms that the proposed colour-correction always leads to improvement.

## Figures and Tables

**Table 1:** Main features of the sets used in the experiments.

**set name**	**main image's characteristics**	**microscope, magnification**	**pixel in saturation**	**vignetting effect**
PANCREAS	Pancreas histology characterized by a very strong vignetting effect	Nikon 10x phase-contrast	present	high

FLY	Fruit fly characterized by a very strong vignetting effect	Optika 4x brightfield	present	high

BONEa	Bone tissue with fully-saturated pixels in the red channel	Nikon 10x brightfield	present	low

BONEb	Bone tissue characterized by a low contrast	Nikon 10x phase-contrast	no present	low

STOMACH	Stomach histology characterized by a low vignetting effect	Optika 4x brightfield	no present	low

TESTICLE	Testicle histology characterized by a low vignetting effect	Optika 4x brightfield	no present	low

MSC	Living mesenchymal stem cells characterized by a low contrast	Nikon 10x phase-contrast	no present	low

**Table 2:** Quantitative analysis of mosaic's quality: RMSE.

***RMSE values***	*mosaic typology*				
*set name*	**TA**	**TB**	**TC**	**TD**	**TE**

**PANCREAS**	25.74	9.17	**8.32**	18.80	*7.20*

**FLY**	12.12	4.66	**4.64**	6.80	*2.81*

**BONEa**	11.43	6.08	6.06	**5.43**	*2.24*

**BONEb**	4.49	1.84	**1.84**	2.79	*1.11*

**STOMACH**	8.71	6.11	6.11	**5.67**	*3.61*

**TESTICLE**	10.59	6.78	6.78	**6.62**	*3.63*

**MSC**	5.06	2.28	**2.28**	3.07	*1.39*

**Table 3:** Quantitative analysis of mosaic's quality: SNR.

***SNR values***	*mosaic typology*				

*set name*	**TA**	**TB**	**TC**	**TD**	**TE**

**PANCREAS**	16.82	25.25	**26.07**	18.94	*27.33*

**FLY**	16.97	25.71	**25.73**	22.53	*29.99*

**BONEa**	25.72	31.17	31.20	**32.39**	*39.89*

**BONEb**	29.94	37.60	**37.60**	33.96	*41.94*

**STOMACH**	25.23	28.18	28.18	**28.84**	*32.72*

**TESTICLE**	23.10	27.01	27.01	**27.24**	*32.45*

**MSC**	27.95	34.71	**34.71**	32.13	*39.01*

**Table 4:** Quantitative analysis of mosaic's quality: UQI.

***UQI values***	*mosaic typology*				

*set name*	**TA**	**TB**	**TC**	**TD**	**TE**

**PANCREAS**	0.9090	0.9789	**0.9825**	0.9451	*0.9869*

**FLY**	0.9822	0.9973	**0.9973**	0.9946	*0.9990*

**BONEa**	0.9678	0.9704	0.9708	**0.9900**	*0.9967*

**BONEb**	0.8930	0.9301	0.9301	**0.9351**	*0.9751*

**STOMACH**	0.9939	0.9961	0.9961	**0.9973**	*0.9986*

**TESTICLE**	0.9839	0.9937	0.9937	**0.9944**	*0.9982*

**MSC**	0.8500	0.8589	0.8589	**0.8923**	*0.9438*

## Data Availability

*MicroMos* source code and standalone executable versions, as well as all the image datasets used in the experiments, are freely available at http://sourceforge.net/p/micromos.

## Supplementary Materials

Supplementary Materials Supplementary Figure 1: TB and TE mosaics. Together with those reported in Figure 8, these mosaics give a whole overview of the sets used in the experiments.
