# Supplementary material for: Colour Vignetting Correction for Microscopy Image Mosaics Used for Quantitative Analyses
Source: Biomed Res Int. 2018 Jun 7;2018:7082154. doi: 10.1155/2018/7082154 (PMC6011154; doi:10.1155/2018/7082154)

Supplementary Materials

Supplementary Figure 1: TB and TE mosaics. Together with those reported in **Figure 8**, these mosaics give a whole overview of the sets used in the experiments.

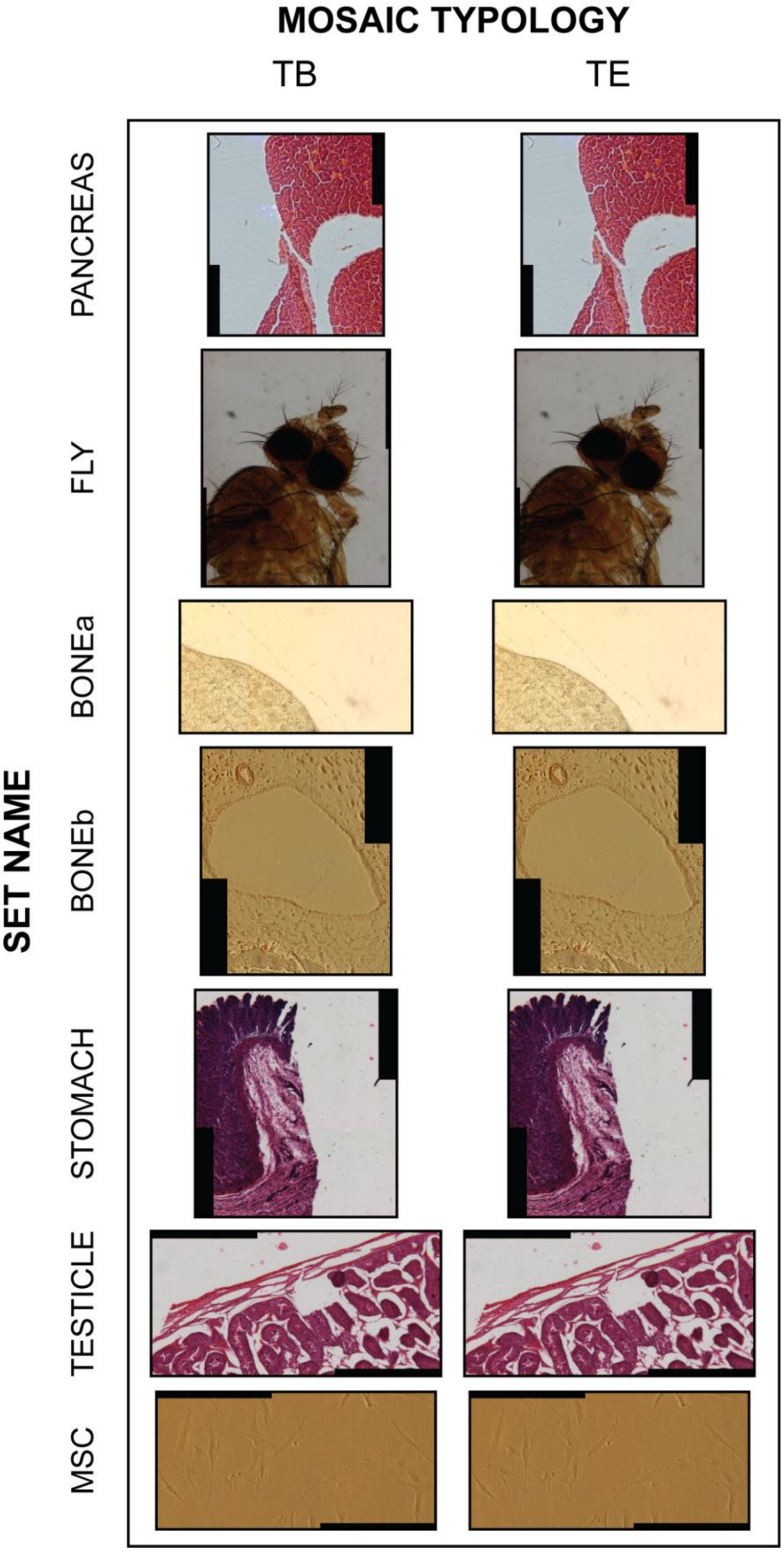

Supplement: Supplementary Materials — Supplementary Figure 1: TB and TE mosaics. Together with those reported in Figure 8, these mosaics give a whole overview of the sets used in the experiments. [file 7082154.f1.pdf]
